# The Contribution of DNA Metabarcoding to Fungal Conservation: Diversity Assessment, Habitat Partitioning and Mapping Red-Listed Fungi in Protected Coastal *Salix repens* Communities in the Netherlands

**DOI:** 10.1371/journal.pone.0099852

**Published:** 2014-06-17

**Authors:** József Geml, Barbara Gravendeel, Kristiaan J. van der Gaag, Manon Neilen, Youri Lammers, Niels Raes, Tatiana A. Semenova, Peter de Knijff, Machiel E. Noordeloos

**Affiliations:** 1 Naturalis Biodiversity Center, Leiden, The Netherlands; 2 Faculty of Science, Leiden University, Leiden, The Netherlands; 3 University of Applied Sciences Leiden, Leiden, The Netherlands; 4 Forensic Laboratory for DNA Research, Human Genetics, Leiden University Medical Centre, Leiden, The Netherlands; Nanjing Agricultural University, China

## Abstract

Western European coastal sand dunes are highly important for nature conservation. Communities of the creeping willow (*Salix repens*) represent one of the most characteristic and diverse vegetation types in the dunes. We report here the results of the first kingdom-wide fungal diversity assessment in *S. repens* coastal dune vegetation. We carried out massively parallel pyrosequencing of ITS rDNA from soil samples taken at ten sites in an extended area of joined nature reserves located along the North Sea coast of the Netherlands, representing habitats with varying soil pH and moisture levels. Fungal communities in *Salix repens* beds are highly diverse and we detected 1211 non-singleton fungal 97% sequence similarity OTUs after analyzing 688,434 ITS2 rDNA sequences. Our comparison along a north-south transect indicated strong correlation between soil pH and fungal community composition. The total fungal richness and the number OTUs of most fungal taxonomic groups negatively correlated with higher soil pH, with some exceptions. With regard to ecological groups, dark-septate endophytic fungi were more diverse in acidic soils, ectomycorrhizal fungi were represented by more OTUs in calcareous sites, while detected arbuscular mycorrhizal genera fungi showed opposing trends regarding pH. Furthermore, we detected numerous red listed species in our samples often from previously unknown locations, indicating that some of the fungal species currently considered rare may be more abundant in Dutch *S. repens* communities than previously thought.

## Introduction

Fungi represent one of the largest groups of living organisms. However, fungi are still poorly understood and appreciated compared to plants and animals and our knowledge of fungal diversity lags far behind [1]. Approximately 100,000 species of Eumycota (the true Fungi) have been described. Already well before the routine use of DNA sequencing in fungal biodiversity assessments, their true diversity was estimated to be around 1.5 million species [2], while more recent estimates suggests there may be 0.7 to 5 million species [3],[4]. Despite the differences among various estimates, it is clear that we currently know only a fraction of the total fungal biodiversity.

The task of discovering a significant portion of yet unknown species has only recently become possible with the advent of high-throughput DNA sequencing of environmental samples that has enormous potential to further boost data acquisition in biodiversity research [5],[6],[7],[8],[9],[10],[11],[12],[13],[14],[15],[16]. This methodology involves identification of multiple species from environmental samples using targeted loci specifically selected with the purpose of identification. These loci are typically the same ones that are used in specimen-based large-scale DNA barcoding efforts (e.g., [17],[18]), hence providing both appropriate power of resolution for the group of interest and maximum amount of reference sequences available from vouchered specimens.

The fungal diversity assessment presented here focuses on *Salix repens* L. sand dune communities along the North Sea coast, because these areas are highly important for nature conservation, water resource management, and recreational purposes. Coastal sand dunes include an outstanding ecological diversity in terms of environmental heterogeneity and species composition [19]. In the Netherlands, they are home to more than half of all higher plant species, in spite of covering only 1% of the land area [20],[21]. In Europe, coastal dune areas have been reduced by ca. 70% in the last century [22], and biodiversity in the remaining dune ecosystems is highly endangered [23]. In the Netherlands, however, coastal dunes have been relatively well preserved, with less than 10% area loss, largely due to their roles in drinking water management and as barriers against the sea [21]. These dunes represent one of the last nutrient-poor habitats in the Netherlands and most plant species featured on the Dutch Red List now have their main distribution in these coastal areas. Moreover, the reserves featured in this manuscript are part of Natura 2000, an ecological network of protected areas in the territory of the European Union (http://www.natura2000.nl/pages/kaartpagina.aspx).

The creeping willow (*Salix repens*) is a widespread shrub in western Europe and inhabits a wide range of vegetation types. It forms both ectomycorrhiza and arbuscular mycorrhiza [24],[25], often supporting a diverse community of symbiotic fungi in its extensive root system [26]. It is a particularly characteristic component of coastal sand dune ecosystems where it is often the only ectomycorrhizal (ECM) host plant [27],[26]. Therefore, it has been repeatedly noted that the presence of *S. repens* adds wealth to the overall fungal diversity in sand dune ecosystems [25],[26]. Somewhat surprisingly, species composition of fungi in *S. repens* communities have not been previously investigated using molecular methods to our knowledge. To date, characterizations of fungi in *S. repens* assemblages have been based on morphology-based identification of fruitbodies and ECM root structures. For example, in one of the early studies comparing above- and below-ground diversity of ECM fungi, Van der Heijden *et al.* [25] found fruitbodies of 78 ECM taxa belonging to 12 genera and identified fifteen ECM root morphotypes in *S. repens* communities in the Netherlands. Watling [26] reported almost 80 species of macrofungi from *S. repens* beds of the northern Scottish islands based on fruiting records. Despite these outstanding pioneer works, important gaps remain in our knowledge about total fungal diversity in this important ecosystem.

In this study, we collected soil samples at multiple locations in strictly protected coastal areas and conducted a large-scale biodiversity assessment of soil fungi inhabiting the rhizosphere of *S. repens* using pyrosequencing techniques. The aims of this work were 1) to characterize fungal communities found in *S. repens* vegetation; 2) compare the effects of environmental factors (e.g., soil pH, geological district) on communities through multiple sites; and 3) to supplement sporocarp-based mapping data points of rare fungi with soil-based data points. Our results provide the first kingdom-wide fungal diversity assessment in coastal sand dune communities and demonstrate the potential use of high-throughput DNA sequencing of environmental samples to fungal conservation.

## Materials and Methods

### The Study Region

**Figure 1:**
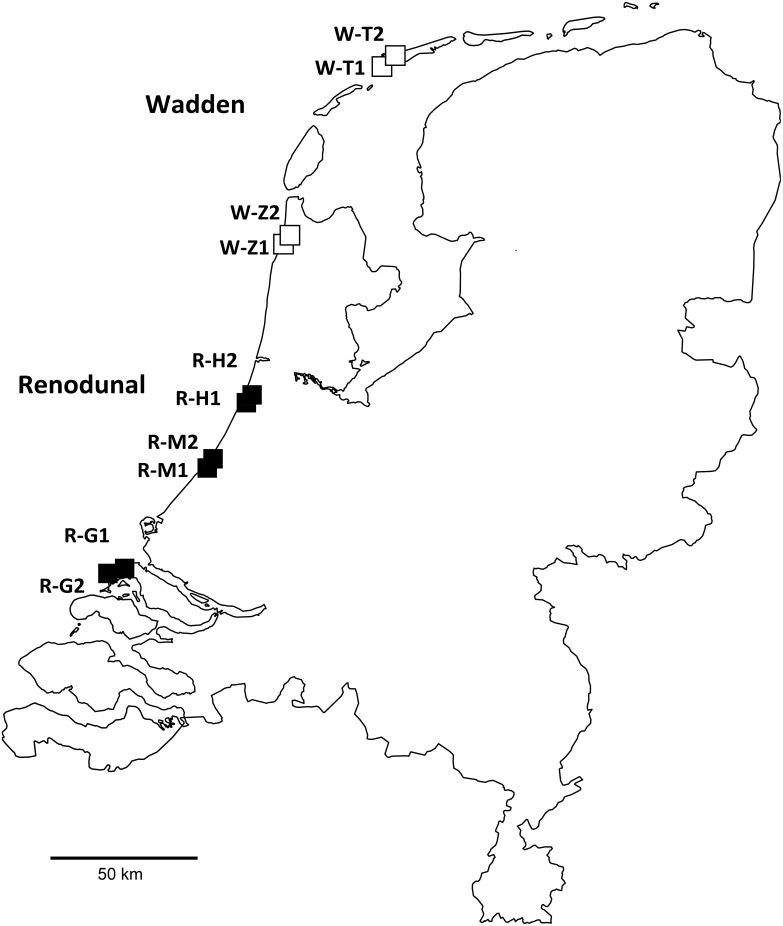
Sampling sites along the coast of the Netherlands. Sites corresponding to the Renodunal and Wadden geological districts are marked with black and white squares, respectively.

The following environmental agencies granted permission for the fieldwork (with sampled field sites): Amsterdamse Waterleidingduinen (Haarlem-Bentveld), Dunea (Wassenaar-Meijendel), Natuurmonumenten (Goeree, Callantsoog-Zwanenwater), and Staatsbosbeheer (Terschelling). GPS coordinates of the sampling sites are listed in Table 1. The field sampling only included soil and did not involve endangered or protected species. The selected sites represented sand dune *S. repens* beds (European Environment Agency habitat code: 2170) in an extended area of joined nature reserves located along the North Sea coast of the Netherlands, representing a south-north transect (Figure 1, Table 1). This coastal dune system is characterized by an oceanic climate with mean annual temperature of 9–10°C (July, 16–17°C; January, 2–3°C) and precipitation of 675–800 mm [21]. Sand-lime content varies between 0.1 and 10% [21], with generally calcareous soils in the southern Renodunal district (represented by the sampling sites Goeree, Meijendel, and Haarlem-Bentveld) and more acidic soils in the northern Wadden district (Zwanenwater, Terschelling). In most areas, the height of the dunes reaches 15–25 m [28]. Current management policies are directed toward multiple purposes, such as drinking water supply, defense against storm floods, recreation, and nature conservation [29]. Soil samples were taken at sites monitored by Natuurmonumenten (Goeree and Zwanenwater), Dunea (Meijendel), Staatsbosbeheer (Terschelling), and the Amsterdamse Waterleidingduinen (Haarlem-Bentveld). In March of 2010, 20 soil cores, 2 cm in diameter and ca. 20 cm deep, were taken at each site in a way that cores were at least 2 m from each other to minimize the probability of sampling the same genet repeatedly. For every plot, the 20 cores were pooled, resulting in a composite soil sample for each plot. Soils were deposited in 50 mL Falcon tubes and stored at –80°C until lyophilization.

**Table 1:** Sampling sites of coastal *Salix repens* communities used in this study.

Site	Code	Geological district, habitat	Latitude, longitude	pH	OTUs
Goeree: Middelduinen	R-G1	Renodunal, wet	51.827238; 3.943405	6.01	971
Goeree: Westduinen	R-G2	Renodunal, wet	51.810850; 3.861609	6.73	934
Wassenaar: Meijendel 1	R-M1	Renodunal, dry	52.137439; 4.341960	7.3	691
Wassenaar: Meijendel 2	R-M2	Renodunal, dry	52.142707; 4.333034	7.42	790
Haarlem-Bentveld: Amsterdamse Waterleidingduinen 1	R-H1	Renodunal, wet	52.338001; 4.531978	7.11	727
Haarlem-Bentveld: Amsterdamse Waterleidingduinen 2	R-H2	Renodunal, dry	52.337312; 4.533910	6.56	660
Callantsoog: Zwanenwater 1	W-Z1	Wadden, dry	52.822073; 4.709229	5.71	867
Callantsoog: Zwanenwater 2	W-Z2	Wadden, wet	52.821516; 4.708264	5.32	762
Terschelling: Groene Strand	W-T1	Wadden, wet	53.364741; 5.201769	6.39	718
Terschelling: West aan Zee	W-T2	Wadden, dry	53.398326; 5.269489	5.78	804

Code, geological district, habitat type (wet dune slack vs. dry dune side and top), geographic coordinates, soil pH, number of quality-filtered sequences and the number of 97% ITS2 sequence similarity OTUs are displayed for each site.

### DNA Extraction, PCR, and Pyrosequencing

We follow the recently proposed guidelines for standardizing the description of next-generation sequencing datasets in publications as proposed by Nilsson *et al.* [30]. Genomic DNA was extracted from 8 g of lyophilized soil from each composite sample using the Mo Bio Powersoil kit following the manufacturer’s instructions. We used high-throughput tag-encoded 454 GS-FLX amplicon sequencing to assess fungal diversity in the selected plant communities. Amplicon libraries were performed using a combination of tagged primers designed for the variable ITS region, as recommended for the tag-encoded pyrosequencing method [31]. Genomic DNA samples were amplified using the fungal-specific primer pair ITS1F (5′-*X*CTTGGTCATTTAGAGGAAGTAA) and ITS4 (5′-*Yxxxxx*TCCTCCGCTTATTGATATGC), where *X* and *Y* represent the two pyrosequencing primers (CCTATCCCCTGTGTGCCTTGGCAGTCTCAGT and CCATCTCATCCCTGCGTGTCTCCGACTCAGA) and *xxxxx* represents the barcodes designed for sample identification. Barcodes differed from all other barcodes by at least two nucleotides. Four PCR reactions were performed per sample using the following temperature program: 95°C/2 min, 25 cycles of 95°C/30 s, 54°C/1 min, 72°C/2 min, and 72°C/10 min. One µl of DNA template was used for the 40 µl PCR reaction containing 25.6 µl of MQ water, 4 µl of 10x buffer, 1.5 µl dNTP’s (2.5 mM), 1.5 µl of reverse and forward primers (10 mM), 4 µl MgCl_2_ (50 mM), 0.5 µl BSA (10 mg/ml) and 0.4 µl BIOTAQ polymerase (5 U/µl). PCR products were pooled for each sample, purified and sequencing-adapters/tags were integrated via MegaPrimers (PCR). The amplicon length and concentration were estimated, and an equimolar mix of all amplicon libraries was used for pyrosequencing (with the ITS4 primer). Tagged samples were pooled and sequenced using the Genome Sequencer GS FLX 454 System (454 Life Sciences/Roche Applied Biosystems, Nutley, NJ, USA) at GATC Biotech AG (Konstanz, Germany) and the Leiden Genome Technology Center (Leiden, the Netherlands).

### Bioinformatic Work

Pyrosequencing resulted in 688 434 reads following quality checks and trimming, with median read length of 284 bp. The raw data have been submitted to the European Nucleotide Archive (ERP001713). We used a parallel version of MOTHUR v. 1.32.1 [32] installed at the University of Alaska Life Sciences Informatics Portal for sequence analyses. We filtered sequences by quality score with a sliding window approach as in Brown *et al.* [33]. The FASTQ files were converted to FASTA and QUAL files, and the sequences were subjected to quality filtering whereby each sequence was screened for thresholds for average Phred score of Q≥25 in a sliding window of 50 bp (qwindowaverage = 25, qwindowsize = 50), no ambiguous bases (maxambig = 0) and homopolymers no longer than 8 bp (maxhomop = 8). Sequences shorter than 150 bp or longer than 400 bp were omitted from further analyses (mintlength = 150, maxlength = 400). A total of 369 181 sequences remained after quality filtering and trimming. Because next-generation sequencing libraries generally vary in size, we normalized the number of sequences for all samples prior to OTU clustering, as recommended by Gihring *et al.* [34], to ensure that estimators across all samples are comparable. For this purpose, we randomly subsampled the number of trimmed and quality-filtered reads to the size of the smallest library (30 497).

The resulting 304 970 sequences served as input for operational taxonomic unit (OTU) clustering using OTUPIPE [35] based on 97% sequence similarity. Such 97% sequence similarity cut-off value is routinely used in fungal community studies as a proxy for species, accounting for intraspecific variation and errors generated by PCR (e.g., [36],[37],[38],[7],[39],[40],[9],[12],[15],[41]. We simultaneously removed 13 279 putatively chimeric sequences, using a curated dataset of fungal ITS sequences as reference dataset [42]. Pyrosequencing datasets comprising orders of magnitude more sequences per taxa than traditional methods tend to contain a higher proportion of artifactual OTUs, because sequencing errors tend to be unique, while the accumulation of real taxa starts to level off [43],[12]. Because most pyrosequencing singletons tend to be artifactual and such sequencing errors can overestimate the diversity of ‘rare taxa’ (e.g., [44],[12]), we opted to be conservative and excluded all singletons from further analyses. For identification, sequences were subjected to USEARCH [45] against the latest release of quality-checked UNITE+INSD fungal ITS sequence database containing both identified and unidentified sequences, many of which are assigned to Species Hypothesis groups as defined by experts using phylogenetic evaluations [46]. OTUs that did not have at least 80% similarity over at least 100 bp to any fungal sequence were excluded from further analyses. Taxonomic affiliations were based on the current Index Fungorum classification as implemented in UNITE.

### Comparing Fungal Communities among Sampling Sites

We used the 97% ITS sequence similarity OTUs inferred earlier as input data for the various ordination methods. To visualize the variation in the occurrence of OTUs across sites, we carried out ordination using non-metric multidimensional scaling (NMDS) with the quantitative version of Sorenson similarity (Bray-Curtis index) with 500 iterations in PC-Ord [47]. Read abundance in 454 sequencing has been shown to be approximately quantitative only within species, while between-species comparisons can be biased by innate sequence structure [39]. Because of such uncertainties regarding the reliability of read abundance, we carried out the ordination analyses with square-root transformed abundance to moderate the influence of OTUs with high sequence counts, while maintaining some approximation of abundance that often reflects ecological significance. The solution with the lowest stress was derived from 250 runs using real data and was then subjected to 250 randomized runs using a Monte Carlo test to evaluate the probability of final the NMDS patterns being greater than chance occurrences. Orthogonal rotation of the resulting NMDS solution was used to maximize correlation between the strongest environmental variables (as indicated by Pearson *r* values) and major axes. The solution with the lowest number of dimensions was selected when the decrease in the final stress was <5 by adding another dimension [47]. The Pearson correlation coefficient (*r*) values between environmental and fungal community variables and axes 1 and 2 were calculated. Soil pH and the number of OTUs representing various taxonomic groups per site were included in a site environmental factor matrix as quantitative variables. For this latter, we used only taxonomic orders and genera represented by at least 3 OTUs were included for a site as quantitative variables (see Table 2 for full list). Geological district (Renodunal = 1, Wadden = 0) was included as categorical variable. Following NMDS ordination of sites, we examined the Pearson correlation values between the community ordination axes and these variables. We also tested whether fungal communities were statistically different across the sites using a multiresponse permutation procedure (MRPP), also in PC-Ord. For the MRPP analyses, three categorical variables were tested: geological district, as described above, habitat type (wet dune slack vs. dry dune side and top), and soil pH (acidic = 0, non-acidic = 1, based on a pH = 6.5 threshold following the USDA soil classification for acidic and non-acidic soils). Finally, we determined any preferences of individual OTUs for pH categories using indicator species analyses.

**Table 2:** Pearson’s correlation values (*r*) for variables in the NMDS analyses.

	Axis 1	Axis 2		Axis 1	Axis 2
	*r*	*r^2^*	*r*	*r^2^*		*r*	*r^2^*	*r*	*r^2^*
**pH**	**0.718**	0.516	0.094	0.009	Ascomycete genera				
OTU richness	−0.263	0.069	−0.29	0.084	*Acremonium*	−0.229	0.052	0.11	0.012
Ascomycota	0.028	0.001	0.306	0.093	***Alternaria***	**0.594**	0.353	−0.144	0.021
Basidiomycota	0.321	0.103	0.503	0.253	***Archaeorhizomyces***	−**0.704**	0.495	0.479	0.229
Glomeromycota	0.33	0.109	0.197	0.039	***Cadophora***	**0.655**	0.429	−0.142	0.02
Mucoromycotina	−0.318	0.101	0.529	0.28	***Cladonia***	**0.854**	0.729	−**0.527**	0.278
**Chytridiomycota**	−**0.585**	0.342	0.159	0.025	*Devriesia*	−0.198	0.039	0.027	0.001
					***Exophiala***	**0.881**	0.777	−0.406	0.165
Ascomycete orders					*Fusarium*	−0.026	0.001	−0.006	0
**Archaeorhizomycetales**	−**0.704**	0.495	0.479	0.229	*Geoglossum*	0.062	0.004	0.176	0.031
Capnodiales	−0.232	0.054	0.222	0.049	*Geomyces*	0.424	0.179	−0.463	0.214
Chaetosphaeriales	−0.371	0.138	0.223	0.05	***Geopora***	**0.73**	0.532	−0.092	0.008
Chaetothyriales	0.242	0.059	−0.282	0.079	*Ilyonectria*	0.344	0.119	−0.213	0.046
Diaporthales	0.202	0.041	−0.47	0.221	***Lachnum***	−**0.854**	0.729	**0.527**	0.278
**Eurotiales**	−**0.599**	0.359	−0.07	0.005	*Lophiostoma*	0.154	0.024	0.199	0.04
Geoglossales	0.012	0	0.258	0.067	*Massarina*	0.02	0	0.107	0.011
Glomerales	0.418	0.175	−0.254	0.065	*Meliniomyces*	−0.392	0.154	0.195	0.038
**Helotiales**	−**0.624**	0.389	−0.058	0.003	***Metarhizium***	0.437	0.191	−**0.59**	0.348
Hypocreales	−0.274	0.075	−0.177	0.031	*Myrothecium*	0.287	0.083	0.155	0.024
**Lecanorales**	**0.829**	0.687	−0.441	0.194	***Penicillium***	−**0.63**	0.397	0.013	0
Microascales	0.195	0.038	−0.257	0.066	*Phialophora*	0.17	0.029	−0.26	0.068
**Onygenales**	**0.643**	0.414	−**0.749**	0.561	***Phoma***	**0.899**	0.808	−0.309	0.096
Orbiliales	−0.396	0.157	0.17	0.029	*Pochonia*	−0.359	0.129	−0.332	0.11
**Pezizales**	**0.921**	0.848	−0.15	0.023	***Podospora***	**0.873**	0.761	−**0.566**	0.32
Phyllachorales	−0.419	0.176	0.014	0	*Preussia*	0.04	0.002	0.167	0.028
**Pleosporales**	**0.759**	0.577	−0.184	0.034	***Scolecobasidium***	**0.588**	0.346	−0.414	0.171
**Saccharomycetales**	−**0.563**	0.317	−0.272	0.074	***Teratosphaeria***	**0.817**	0.667	−0.211	0.044
**Sordariales**	**0.883**	0.779	−0.492	0.242	*Trichoderma*	−0.378	0.143	0.051	0.003
**Verrucariales**	−**0.535**	0.286	0.485	0.235	***Verticillium***	−**0.551**	0.303	−0.435	0.189
**Xylariales**	−0.037	0.001	**0.57**	0.325					
Basidiomycete orders					Basidiomycete genera				
Agaricales	0.479	0.229	−0.137	0.019	*Ceratobasidium*	−0.255	0.065	−0.331	0.11
**Auriculariales**	−**0.796**	0.634	0.122	0.015	*Clavicorona*	−0.445	0.198	−0.118	0.014
Boletales	0.015	0	0.213	0.045	***Clavulina***	−**0.63**	0.397	0.052	0.003
Cantharellales	−0.179	0.032	−0.292	0.085	***Clavulinopsis***	−**0.564**	0.318	0.125	0.016
Corticiales	−0.186	0.035	−0.19	0.036	***Cortinarius***	−**0.62**	0.385	**0.594**	0.353
Filobasidiales	−0.398	0.158	−0.023	0.001	*Cryptococcus*	−0.183	0.034	−0.32	0.102
Hymenochaetales	−0.465	0.216	−0.208	0.043	***Entoloma***	−0.453	0.205	**0.672**	0.451
Polyporales	−0.212	0.045	−0.376	0.142	***Hebeloma***	−0.089	0.008	**0.503**	0.253
Russulales	−0.447	0.2	0.026	0.001	*Hygrocybe*	0.166	0.028	−0.126	0.016
Sebacinales	0.39	0.152	−0.13	0.017	***Inocybe***	**0.836**	0.698	−0.164	0.027
Sporidiobolales	−0.372	0.138	0.122	0.015	***Laccaria***	−0.489	0.239	**0.52**	0.27
**Thelephorales**	**0.824**	0.679	−0.456	0.208	***Mycena***	−**0.56**	0.314	−0.165	0.027
**Trechisporales**	−**0.752**	0.566	−0.129	0.017	***Panaeolus***	**0.745**	0.555	−0.216	0.047
Tremellales	0.069	0.005	0.106	0.011	*Paxillus*	−0.114	0.013	0.255	0.065
**Trichosporonales**	−**0.853**	0.727	0.243	0.059	*Rhodotorula*	−0.416	0.173	−0.036	0.001
					*Rickenella*	−0.465	0.216	−0.208	0.043
					*Russula*	−0.477	0.227	0.471	0.221
					*Sebacina*	−0.115	0.013	0.288	0.083
					*Sporobolomyces*	−0.004	0	0.265	0.07
					*Stropharia*	0.356	0.127	−0.026	0.001
					***Thanatephorus***	**0.515**	0.265	−0.237	0.056
					***Thelephora***	−**0.633**	0.4	−0.223	0.05
					***Tomentella***	**0.939**	0.882	−0.403	0.163
					***Trichosporon***	−**0.853**	0.727	0.243	0.059

Ordinations were performed with square-root transformed abundance in the OTU vs. site matrix. Final stress value for the 2-dimensional NMDS solution was 0.03062. Variables with |*r|* ≥0.5 values are shown in bold and are displayed in the NMDS ordinations in Figure 3.

## Results

### OTU Diversity and Taxonomic Affiliations

Out of the 688 434 original reads, 402 500 sequences passed the various filtering steps. After normalizing the library size across all samples, 304 970 sequences were assembled into 7156 OTUs of which 1788 were singletons. After excluding all singletons and OTUs with <80% similarity or <100 bp pairwise alignment length to a fungal sequence as performed by USEARCH, 3611 OTUs were retained for subsequent analyses. The number of OTUs at a given site ranged from 660 (Amsterdamse Waterleidingduinen, R-H2) to 971 (Goeree: Middelduinen, R-G1) (Table 1).

**Figure 2:**
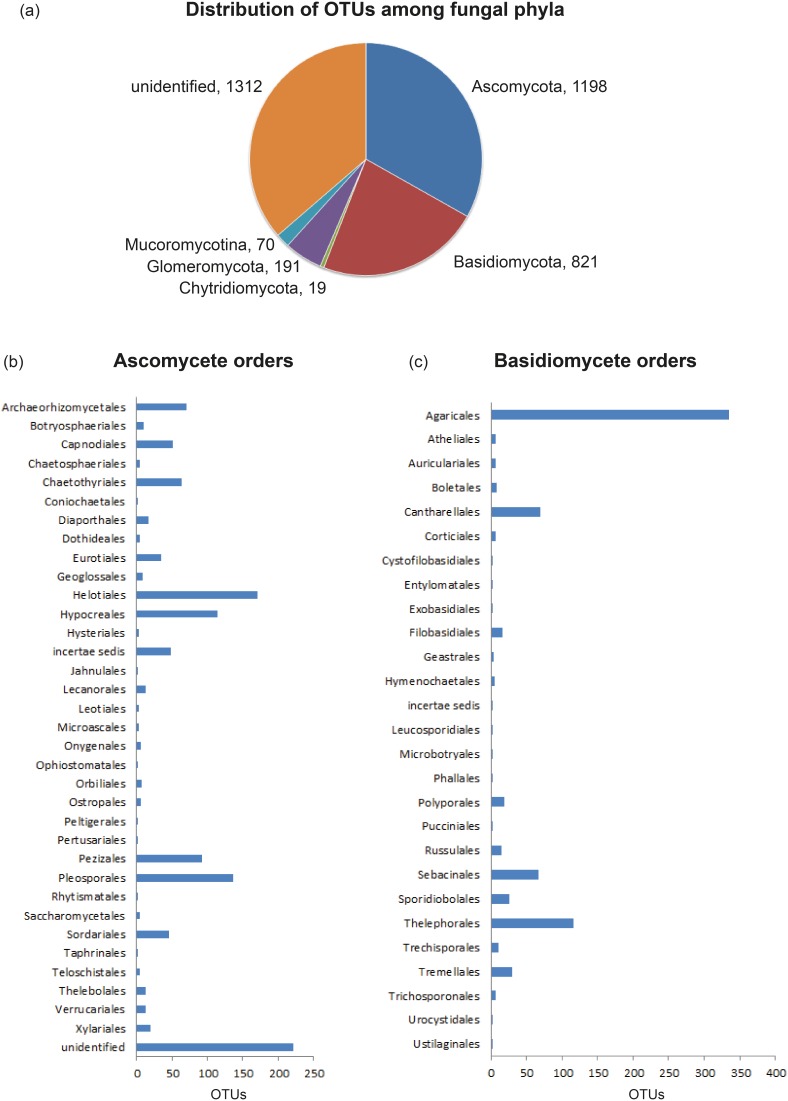
Taxonomic groupings of fungi retrieved from the samples. Proportional distribution of the 1022 unique BLASTN hits among different phyla and taxonomic orders in Basidiomycota and Ascomycota across all sampling sites based on sequences most closely related to 97% ITS OTUs in our sample.

Ascomycota was the dominant phylum and accounted for 33.18% of the OTUs, followed by Basidiomycota (22.73%) Glomeromycota (5.29%), Mucoromycotina, *incertae sedis* (1.94%), and Chytridiomycota (0.53%), while unidentified fungal OTUs with most similar sequences to other environmental sequences without assignment to phylum accounted for 36.33% (Figure 2a). In Ascomycota, the ranking of taxonomic orders based on the number of representative OTUs was as follows: Helotiales (171), Pleosporales (136), Hypocreales (114), Pezizales (92), Archaeorhizomycetales (71), Chaetothyriales (64), Capnodiales (51), Sordariales (45), Eurotiales (34), Xylariales (19), Diaporthales (17), Verrucariales (13), Lecanorales (13), Thelebolales (12), Botryopshpaeriales (10), followed by numerous other orders with <10 OTUs. In addition, there were 49 ascomycete OTUs with *incertae sedis* classification, representing mostly mitosporic, asexual fungi (Figure 2b). In Basidiomycota, Agaricales was by far the most species-rich order in the sample with 335 OTUs, followed by Thelephorales (116), Cantharellales (69), Sebacinales (67), Tremellales (30), Sporidiobolales (26), Polyporales (19), Filobasidiales (16), Russulales (15), Trechisporales (11) and numerous other orders with <10 OTUs each, while there were only 2 basidiomycete OTUs with uncertain taxonomic placement (i.e, *incertae sedis*) (Figure 2c).

There were 354 OTUs with >97% sequence similarity to publicly available sequences with species identification (Table S1). Although most of these represented microfungi, we also detected many species that form macroscopic fruitbodies (e.g., mushrooms, bracket fungi, cup fungi, earth tongues, truffles etc.), among which we found numerous red listed species in our samples, often from previously unknown locations, based on mapping data and red list categories obtained from the Fungal Mapping website of the Dutch Mycological Society (http://www.verspreidingsatlas.nl/paddenstoelen).

### Comparing Fungal Communities among Sampling Sites

**Figure 3:**
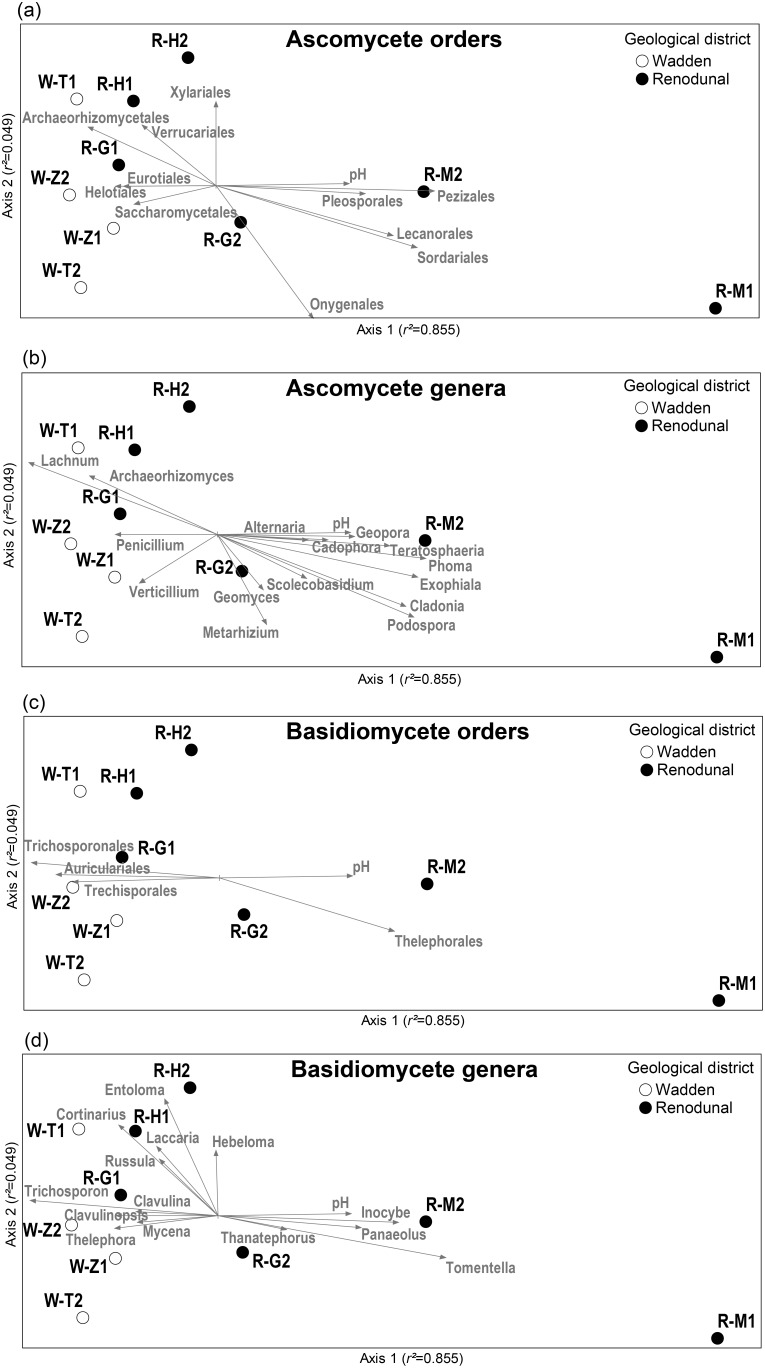
Non-metric multidimensional scaling (NMDS) ordination plot for fungal communities from *Salix repens* sites. Ordinations were based on the square-root transformed abundance of 97% ITS sequence similarity OTUs. Labels, localities and descriptions of the sampling sites are given in Table 1. Vectors are shown for soil pH, total OTU richness, and for the most diverse taxonomic orders (A) and genera (B) that correlated with the ordination axes at *r^2^*>0.200.

NMDS analyses resulted in a 2-dimensional solution with a final stress of 0.03062 and a final instability <0.00001. The Monte Carlo test results indicated that this 2-dimensional solution using the real data was significantly better than chance occurrences (*p* = 0.008). The two axes explained the majority of variability in the sampled fungal communities (axis 1: *r^2^* = 0.855; axis 2: *r^2^* = 0.049; total *r^2^* = 0.904; orthogonality = 79.5%). The NMDS ordination plot was orthogonally rotated by pH to visualize correlations between pH and fungal community composition in general and the taxonomic groups in particular. The Pearson correlation coefficient (*r*) values between all fungal community variables and axes 1 and 2 are displayed in Table 2. As in the fungal community ecology paper of Rogers *et al.* [48], we considered variables with |*r|* ≥0.5 values for axis 1 important for characterizing changes in fungal community structure along a pH gradient and these variables were superimposed on the ordination plot as direction and strength vectors (Figure 3).

There was a strong structuring of the occurrence of fungal OTUs according to soil pH, that co-correlated with the geological district. Sites belonging to the Wadden district were clustered on the left side of the ordination plot with strongly negative axis 1 values. Even though the Renodunal district sites were more scattered, the Wadden and Renodunal sites formed non-overlapping clusters (Figure 3). However, one Renodunal site (Goeree: Middelduinen, R-G1), that had an acidic soil pH value uncharacteristic of the Renodunal sites, was appeared to be more similar to the Wadden communities than to other Renodunal sites. MRPP tests confirmed the importance of soil pH in shaping fungal community composition (effect size *A* = 0.04259, probability *p* = 0.00463), while the effect of geological district was somewhat weaker (*A* = 0.02786, *p* = 0.03157). Habitat type (wet dune slack vs. dry dune side and top) did not seem to have a significant influence on community composition (*A* = 0.01113, *p* = 0.17961).

The joint plots illustrate the strength and direction of correlation of variables to each ordination axis (Figure 3). The correlation of soil pH with axis 1 was strong (*r* = 0.718). At the phylum level, only Chytridiomycota showed correlation with axis1 (*r* = −0.585) and was more diverse in the acidic sites, while diversity in other phyla did not show significant trends. At the taxonomic order level, the ascomycete Archaeorhizomycetales, Helotiales, Eurotiales, Saccharomycetales, and Verrucariales and the basidiomycete Trichosporonales, Auriculariales, Trechisporales were found to be more diverse in the acidic sites (Figure 3, Table 2). Orders showing greater richness in the alkaline soil samples included the ascomycete Pezizales, Sordariales, Lecanorales, Pleosporales, Onygenales and the basidiomycete Thelephorales. At the genus level, the diversity in ascomycete genera *Archaeorhizomyces*, *Lachnum*, *Penicillium*, and *Verticillium* and basidiomycete genera *Trichosporon*, *Thelephora*, *Clavulinopsis*, *Cortinarius*, *Clavulina*, and *Mycena* were negatively correlated with pH, while the ascomycete *Phoma*, *Exophiala*, *Podospora*, *Cladonia*, *Teratospora*, *Geopora*, *Cadophora*, *Alternaria*, *Scolecobasidium* and the basidiomycete *Tomentella*, *Inocybe*, *Panaeolus*, and *Tanatephorus* were more diverse in the alkaline samples (Figure 3, Table 2).

There were 96 OTUs identified as significant (*p*<0.05) indicators for a certain pH category. Of these, 82 were indicators for acidic and 14 for alkaline sites. Several indicators belonged to root-associated fungi. For example, an unidentified root-endophytic *Phialocephala* and the ECM *Russula pascua* (SH117152.05FU), *Thelephora albomarginata*, *Thelephora* sp. (SH112629.05FU), and *Tulasnella* sp. (SH117022.05FU) were associated with acidic sites, while the ECM *Inocybe dunensis* (SH110718.05FU) and an unidentified *Tomentella* were characteristic of the alkaline sites. In addition, there were several saprobic that were found almost exclusively in the acidic soil samples, such as *Cryptococcus* sp. (SH117277.05FU), *Fusarium* sp. (SH106152.05FU), various *Mortierella* species, *Mycena simian* (SH102569.05FU), *Penicillium adametzii*, *Rickenella fibula* (SH109302.05FU), *Schizoblastosporion starkeyihenricii*, *Umbelopsis ramanniana* (SH100505.05FU), and a *Verticillium* sp. (SH111596.05FU), some of which can be potentially pathogenic. An unidentified species of the nematode pathogen *Pochonia* genus (SH106625.05FU) also showed strong preference for acidic sites. There were only two saprobic species identified as indicators for the alkaline soils: *Fusarium torulosum* and a *Podospora* species. The full list of indicator OTUs are shown in Table 3.

**Table 3:** OTUs considered as significant indicators of the altitudinal vegetation zones with corresponding *p*-values, and with accession numbers, sequence similarity, pairwise alignment length, name, and taxonomic classification of the most similar sequence in the UNITE+INSD database.

OTU	pH	*p*	Accession no.	%	bp	SH	Name	Phylum	Order
1573	acidic	0.0402	HQ212048	99.1	230	SH114088.05FU	Agaricomycotina sp.	Basidiomycota	-
1200	acidic	0.0084	EF445994	80.4	337	SH113104.05FU	*Arthrobotrys eudermata*	Ascomycota	Orbiliales
339	acidic	0.017	JQ272358	98.1	324	-	Ascomycota sp.	Ascomycota	-
3541	acidic	0.0426	JQ272358	96.5	198	-	Ascomycota sp.	Ascomycota	-
747	acidic	0.0306	JQ272358	99.1	324	-	Ascomycota sp.	Ascomycota	-
1385	acidic	0.047	HF947900	97.1	314	-	Ascomycota sp.	Ascomycota	-
1687	acidic	0.0382	HF947896	95.3	215	-	Ascomycota sp.	Ascomycota	-
397	acidic	0.047	HF947899	100	322	-	Ascomycota sp.	Ascomycota	-
535	acidic	0.0468	HM230880	95.3	257	-	Chaetothyriales	Ascomycota	Chaetothyriales
361	acidic	0.0084	KF060235	100	350	-	*Cryptococcus* sp.	Basidiomycota	*Incertae sedis*
6307	acidic	0.0084	FJ553137	96.5	201	SH117277.05FU	*Cryptococcus* sp.	Basidiomycota	*Incertae sedis*
474	acidic	0.027	JQ437607	100	321	SH106152.05FU	*Fusarium* sp.	Ascomycota	Hypocreales
4597	alkaline	0.023	JX534258	99.2	258	SH107221.05FU	*Fusarium torulosum*	Ascomycota	Hypocreales
3605	acidic	0.0376	HE794041	97.2	181	SH113008.05FU	Glomeraceae sp.	Glomeromycota	Glomerales
347	alkaline	0.0084	JN859279	100	303	SH167718.05FU	Helotiales	Ascomycota	Helotiales
395	acidic	0.0468	HM190115	99.7	327	SH115846.05FU	Helotiales sp.	Ascomycota	Helotiales
4961	acidic	0.0244	JN655622	98.7	227	SH115846.05FU	Helotiales sp.	Ascomycota	Helotiales
686	acidic	0.0486	JN655578	100	320	SH114192.05FU	Helotiales sp.	Ascomycota	Helotiales
746	acidic	0.0476	JN655598	97.9	338	SH117370.05FU	Helotiales sp.	Ascomycota	Helotiales
2760	acidic	0.044	HQ021773	83.6	238	-	Hyaloriaceae sp.	Basidiomycota	Auriculariales
773	acidic	0.044	HQ021773	83.6	281	-	Hyaloriaceae sp.	Basidiomycota	Auriculariales
338	alkaline	0.0426	UDB017616	100	249	SH110718.05FU	*Inocybe dunensis*	Basidiomycota	Agaricales
475	acidic	0.047	JQ272448	100	256	-	*Mortierella macrocystis*	Mucoromycotina	Mortierellales
608	acidic	0.0494	JX976005	99.7	331	-	*Mortierella parvispora*	Mucoromycotina	Mortierellales
577	acidic	0.0494	KC018246	100	198	-	*Mortierella* sp.	Mucoromycotina	Mortierellales
6109	acidic	0.0242	JN943795	85.8	225	SH117207.05FU	*Mortierella verticillata*	Mucoromycotina	Mortierellales
4807	acidic	0.044	GU234138	95	179	SH102569.05FU	*Mycena simia*	Basidiomycota	Agaricales
812	acidic	0.0084	GU234138	98.5	205	SH102569.05FU	*Mycena simia*	Basidiomycota	Agaricales
379	acidic	0.0162	KC773822	99.6	259	-	*Penicillium adametzii*	Ascomycota	Eurotiales
1127	acidic	0.0084	FN678832	99.6	226	SH116036.05FU	Pezizomycetes sp.	Ascomycota	-
781	acidic	0.0084	JQ272412	100	264	-	Pezizomycotina sp.	Ascomycota	-
1249	acidic	0.0188	JQ346810	99.1	338	-	*Phialocephala* sp.	Ascomycota	Helotiales
844	acidic	0.047	EU726301	99.7	322	-	Phyllachorales sp.	Ascomycota	Phyllachorales
821	alkaline	0.0166	KC218448	100	244	-	*Plectosphaerella* sp.	Ascomycota	Incertaesedis
1469	acidic	0.047	DQ516079	98.9	187	SH106625.05FU	*Pochonia* sp.	Ascomycota	Hypocreales
158	alkaline	0.0084	KC180725	100	154	-	*Podospora* sp.	Ascomycota	Sordariales
859	acidic	0.047	UDB015270	99.3	272	SH109302.05FU	*Rickenella fibula*	Basidiomycota	Hymenochaetales
1456	acidic	0.0468	FN687605	95.7	161	-	*Russula pascua*	Basidiomycota	Russulales
627	acidic	0.0468	UDB017659	98.5	197	SH117152.05FU	*Russula pascua*	Basidiomycota	Russulales
429	acidic	0.0294	HF558658	99.6	262	-	*Schizoblastosporion starkeyihenricii*	Ascomycota	Saccharomycetales
1618	alkaline	0.0294	JN802314	98.1	210	SH113075.05FU	Sordariales sp.	Ascomycota	Sordariales
346	acidic	0.0084	JQ761571	99	196	SH112969.05FU	Sordariomycetes sp.	Ascomycota	-
555	acidic	0.023	HQ211703	91.6	323	SH100328.05FU	Sordariomycetes sp.	Ascomycota	-
560	acidic	0.047	UDB017379	100	216	-	*Thelephora albomarginata*	Basidiomycota	Thelephorales
514	acidic	0.047	JN858076	100	192	SH112629.05FU	*Thelephora* sp.	Basidiomycota	Thelephorales
1281	alkaline	0.0458	KC840637	99.1	222	-	*Tomentella* sp.	Basidiomycota	Thelephorales
568	acidic	0.0494	GQ268679	88.4	258	-	Trechisporales	Basidiomycota	Trechisporales
1126	acidic	0.0376	JF691365	96.2	213	SH110459.05FU	Trechisporales sp.	Basidiomycota	Trechisporales
420	acidic	0.0426	JF519135	100	195	SH114084.05FU	Trechisporales sp.	Basidiomycota	Trechisporales
5679	acidic	0.044	JF519135	95.5	156	SH114084.05FU	Trechisporales sp.	Basidiomycota	Trechisporales
4735	acidic	0.0234	JQ272445	96.5	173	-	Tremellomycetes sp.	Basidiomycota	-
1929	acidic	0.047	JN655663	99.2	242	SH117022.05FU	*Tulasnella* sp.	Basidiomycota	Cantharellales
2522	acidic	0.0494	EU715662	97.4	193	SH100505.05FU	*Umbelopsis ramanniana*	Mucoromycotina	Mucorales
540	acidic	0.0252	KC489481	96.4	195	-	*Umbelopsis* sp.	Mucoromycotina	Mucorales
561	acidic	0.0166	HQ157863	98.4	253	SH102024.05FU	*Umbelopsis* sp.	Mucoromycotina	Mucorales
731	acidic	0.033	KC489481	99	204	-	*Umbelopsis* sp.	Mucoromycotina	Mucorales
1205	acidic	0.047	DQ914739	97.4	229	SH111596.05FU	*Verticillium* sp.	Ascomycota	*Incertae sedis*
1037	acidic	0.044	KC588535	89.4	227	-	uncultured fungus	-	-
1076	acidic	0.0084	KC588535	86.5	296	-	uncultured fungus	-	-
1211	alkaline	0.049	AJ875364	99.7	343	SH100236.05FU	uncultured fungus	-	-
1459	acidic	0.0084	JQ312779	100	209	-	uncultured fungus	-	-
1651	acidic	0.047	HM439535	96.8	220	SH101693.05FU	uncultured fungus	-	-
1845	acidic	0.0084	FJ197879	93.1	232	-	uncultured fungus	-	-
1913	acidic	0.047	EF521253	80.6	345	-	uncultured fungus	-	-
1969	acidic	0.047	JQ313102	99.6	226	-	uncultured fungus	-	-
2667	alkaline	0.049	EU516756	89.6	328	-	uncultured fungus	-	-
328	acidic	0.0464	JQ312820	98.5	259	-	uncultured fungus	-	-
358	alkaline	0.0458	JN889968	99.4	315	SH102623.05FU	uncultured fungus	-	-
363	acidic	0.0084	KC588722	100	261	-	uncultured fungus	-	-
367	alkaline	0.0084	JX316640	99	306	-	uncultured fungus	-	-
401	acidic	0.0244	KC588704	100	320	-	uncultured fungus	-	-
4316	alkaline	0.0188	GU308427	99.6	255	-	uncultured fungus	-	-
4368	acidic	0.0224	KC416188	97.5	158	-	uncultured fungus	-	-
4423	acidic	0.044	KC416184	95	161	-	uncultured fungus	-	-
451	acidic	0.0084	AF504839	99.7	364	SH109673.05FU	uncultured fungus	-	-
466	alkaline	0.034	FN397289	100	322	SH112415.05FU	uncultured fungus	-	-
4887	acidic	0.0396	JQ312820	91.7	229	-	uncultured fungus	-	-
502	acidic	0.0084	DQ309106	97.6	328	SH114572.05FU	uncultured fungus	-	-
573	acidic	0.047	JQ312957	94.5	218	-	uncultured fungus	-	-
6263	acidic	0.047	JQ312957	96.1	207	-	uncultured fungus	-	-
630	acidic	0.047	AM260886	97.8	274	SH110947.05FU	uncultured fungus	-	-
643	acidic	0.0084	KC588567	99.3	283	-	uncultured fungus	-	-
6519	alkaline	0.049	JX316640	95.7	207	-	uncultured fungus	-	-
6520	acidic	0.048	KC588554	95.9	217	-	uncultured fungus	-	-
665	acidic	0.047	DQ309136	99.6	271	-	uncultured fungus	-	-
711	acidic	0.047	HM136622	99.2	355	-	uncultured fungus	-	-
713	acidic	0.047	JQ312796	99.5	199	-	uncultured fungus	-	-
723	acidic	0.036	DQ093782	97.6	329	SH099908.05FU	uncultured fungus	-	-
762	acidic	0.0458	JQ312820	96.4	193	-	uncultured fungus	-	-
787	acidic	0.0084	JF300531	90.2	368	-	uncultured fungus	-	-
803	acidic	0.0084	JN890270	96.3	327	-	uncultured fungus	-	-
838	acidic	0.0434	FN298702	93.3	326	SH104632.05FU	uncultured fungus	-	-
852	acidic	0.0454	JQ312820	97.8	230	-	uncultured fungus	-	-
857	acidic	0.0162	HM037687	99	196	SH110459.05FU	uncultured fungus	-	-
899	acidic	0.047	HM208720	99.4	334	SH099545.05FU	uncultured fungus	-	-
828	acidic	0.0308	HQ021815	99.7	320	SH108513.05FU	uncultured fungus	-	-

Where available, the Species Hypothesis (SH) numbers are given for the corresponding sequence as published by Köljalg et al. (2013).

## Discussion

### Acidic vs. Non-acidic Sand Dune Fungal Communities

While environmental factors influencing the distribution of plants and animals have been thoroughly studied, environmental factors controlling the spatial patterns and community composition of soil microorganisms are still poorly understood [49]. Recent molecular studies on various microbial groups (mostly bacteria) have started to explore the distributional patterns of soil communities, however, many of them applied DNA-fingerprinting techniques or phospholipid analyses that do not permit the taxonomic surveys and comparisons of different communities [50],[51]. Soil pH is generally implicitly considered important for shaping fungal communities with some supporting evidence from experimental studies mostly with AM fungi [52],[53] and, in recent years, from joint pyrosequencing analyses of bacterial and fungal communities in agricultural soils and land use types [54],[49]. Unfortunately, both of these latter sequencing studies relied on nuclear small-subunit (SSU) ribosomal genes, meaning that their power of taxonomic resolution was at family-level groups at best. Therefore, information on the effect of soil pH on the distribution of specific fungal taxa and on the composition of their communities in natural habitats remains surprisingly scarce.

**Figure 4:**
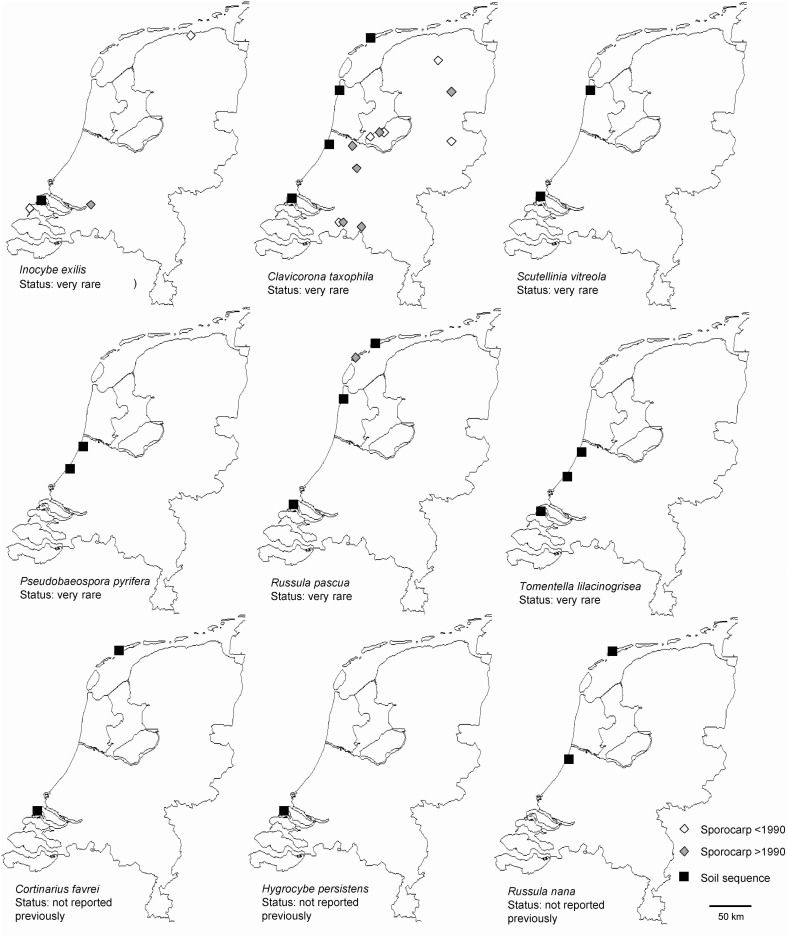
Examples for OTUs found in our soil samples showing high sequence similarity with rare, red-listed fungi. Sporocarp records (before and after 1990) are based on the mapping database of the Dutch Mycological Society (http://www.verspreidingsatlas.nl/paddenstoelen).

Our large-scale comparison of various acidic and non-acidic coastal dune *Salix repens* beds clearly indicates that soil pH strongly correlates with the composition of fungal communities. In NMDS ordinations, soil pH had high Pearson correlation values with the dominant ordination axis and overrode the effect of the geological district (Wadden vs. Renodunal) on community composition in both the NMDS and the MRPP analyses (Figures 3 and 4). This is in agreement with the findings of Van der Heijden *et al*. [25] that soil pH significantly contributed to the variation explained in ECM morphotype composition of communities associated with *S. repens*. In our NMDS ordinations and MRPP analyses, *Inocybe* and *Tomentella* showed strong preference for alkaline soils, while *Clavulina*, *Cortinarius*, *Thelephora*, and to some extent *Laccaria*, were more diverse in acidic sites (Figure 3, Table 2). It is worth noting, that because our study focuses on natural systems, it is impossible to determine based on our current data whether the communities are structured directly by pH or indirectly via the interaction of pH with soil communities and various environmental factors (e.g., nutrient availability, organic C characteristics, vegetation differences etc.). Because results from previous studies using a pH gradient with controlled variables suggest no or only weak effect of pH on community structure [54],[49] and because many fungal species have a relatively wide pH optimum (e.g. [55],[56]), it is likely that the observed correlation of pH with community composition is mainly indirect, e.g., via nutrient availability and altered competitive interactions between soil fungi and bacteria [57], and other soil biota. While our data suggest that the effect of soil pH on fungal community composition may be stronger in relatively undisturbed sites than in agricultural settings, disentangling causal relationships is beyond the scope of our work.

### Root-associated Fungi

Although taxa detected by our sequencing efforts featured groups from a wide variety of ecological guilds, including many saprobic and/or plant pathogenic fungi (e.g., *Fusarium cereale*, *Gibberella zeae, Phoma multirostrata*) as well as animal or fungal pathogens (e.g., *Hypomyces chrysospermus, Metarhizium anisopliae*, *M. flavoviride* and other Hypocreales), we focus our discussion on groups that are most relevant to current conservation priorities, such as fungi with macroscopic fruitbodies and/or with symbiotic associations with *S. repens* or with red listed plants (e.g. orchids) in these habitats.

Previous studies based on sporocarps and ECM root structures have shown that *Salix repens* communities generally harbour numerous ectomycorrhizal basidiomycetes [25],[26]. This was confirmed by our large-scale soil sequence data, as we found 418 ECM OTUs. The majority of these showed similarity to basidiomycete genera known to form associations with willows (e.g. [58],[59],[15]), such as *Inocybe* (95 OTUs), *Thelephora/Tomentella* (83), *Cortinarius* (36), *Hebeloma* (10), *Russula* (7), *Laccaria* (7), *Clavulina* (6), *Lactarius* (5), *Paxillus* (2). Also, there were 4 OTUs (*Amanita pantherina, Hydnum rufescens, Suillus luteus* and *Tulasnella* sp.) that represent species that are known to form ECM associations with other host trees found near the sampling sites, such as oak, pine or poplar. In addition, we found 52 OTUs belonging to the Sebacinaceae, a taxonomically difficult group with diverse ecological roles, including various forms of mycorrhizae (ECM, orchid, ericoid, arbutoid) and other associations with plant roots [60]. We also identified several ectomycorrhizal ascomycetes: *Cenococcum geophilum*, one of the most widespread and abundant ECM fungus with a wide host range [59], *Geopora arenicola* and *G. cervina,* a genus known to occur in sand dunes [61], *Helvella compressa* and *H. maculata*, and an unidentified *Trichophaea* species. In addition, we found four truffles that are likely symbionts of *Quercus* that is widespread in the dunes: *Tuber anniae*, and three unidentified *Tuber* species.

*Salix repens* is one of the very few plants known to form both ECM and AM [24],[62]. In dry coastal foredunes, where phosphorus is the main limiting nutrient, AM fungi and their hosts generally predominate, while nitrogen is the main limiting element in dune slacks, resulting in the dominance of ECM taxa [62]. With the ability to form both ECM and AM associations, *Salix repens* is particularly well-suited to survive in the highly dynamic dune ecosystems and is able to colonize habitats that range from dry to wet and from acidic to calcareous [62]. We detected 191 glomeromycete OTUs in our samples, representing *Glomus* (53), *Rhizophagus* (10), *Archaeospora* (6), *Acaulospora* (4), *Claroideoglomus* (4), *Scutellospora* (4), *Cetraspora* (3), *Paraglomus* (3), *Entrophospora* (2), *Diversispora* (1), *Racocetra* (1), and 100 unidentified glomeromycete OTUs. Because the majority of land plants are known to form AM, it is likely that the majority of these taxa are also associated with a wide range of plants beside *Salix repens*.

We also found numerous OTUs with very high sequence similarity to known dark septate root endophytes (DSE) (e.g., *Cadophora, Cladophialophora*, *Exophiala, Leptodontidium, Meliniomyces*, *Phialocephala*, *Phialophora* spp.). Although DSE colonization and diversity have previously been shown to be higher in acidic conditions in general [63],[64], our data suggest that different genera may prefer different pH conditions. For example, *Cadophora* and *Exophiala* were represented by more OTUs in the alkaline sites, while *Meliniomyces* was somewhat more diverse in samples with lower pH (Table 2).

The sampling areas included in our study harbor some of the richest orchid populations in the Netherlands. The diversity of orchid mycorrhizae may be particularly relevant for nature conservation as they provide symbioses to this unique group of plants with high public appeal and conservation value. The majority of orchid mycorrhizal symbionts belong to the Ceratobasidiaceae, Sebacinaceae and Tulasnellaceae [65],[66], although ECM fungi have also been reported to be associated with orchids, albeit mainly with non-photosynthetic, mycoheterotrophic species that often use the ECM fungus as a channel to obtain carbohydrates from the ECM host tree [65],[67]. Among the first group of orchid symbionts that are typically associated with photosynthetic orchids, we found 21 Ceratobasidiaceae and 12 Tulasnellaceae OTUs in addition to the 52 Sebacinaceae OTUs mentioned above. Among other ECM lineages, the basidiomycete *Inocybe, Tomentella*, and the ascomycete *Tuber* species have been found to form mycorrhizal associations with the mycoheterotrophic orchid *Epipactis helleborine* [67],[68]. This orchid is represented by two subspecies in the Netherlands, among these, *E. h. ssp. helleborine* is widespread in a variety of habitats, while *E. h. ssp. neerlandica* is restricted to the dunes and is endangered [68]. This latter rare subspecies is present in most of our sampling sites and we found multiple species of all three ECM genera reportedly associated with *E. helleborine*. *Tomentella* and *Inocybe* were the most species-rich genera in our samples and are important symbionts for *Salix repens* as well, as specified earlier. Because *E. h. ssp. neerlandica* is mostly found near *Salix repens* [68],[69], it is highly likely that willow-associated ECM fungi are crucial for the survival of this endangered orchid. The same might apply to two other rare orchid species in the Netherlands, *Platanthera bifolia* and *Dactylorhiza incarnata* ssp. *coccinea* of which the associated ECM fungi are still largely unexplored but which are also mostly found near *Salix repens* [69].

### Contribution of Soil Data to Mapping Rare Fungi

The Netherlands boasts one of the most efficient programs in the world for gathering distribution data on macrofungi, where the Netherlands Mushroom Mapping Working Group (Werkgroep Paddenstoelenkartering Nederland, WPN) has been producing distribution maps and fruiting statistics since 1980 with a nation-wide network of ca. 600 volunteers that produce appr. 60,000 data points yearly [70],[71],[72]. The long-term mapping program of the WPN has been crucially important in compiling a near complete checklist of macrofungi [73], in highlighting high diversity sites [70], and in drawing attention to the serious decline of macrofungi in the Netherlands [72]. As a result, an official national Red List of fungi was published in 1996, followed by a revision in 2008 [74]. On the other hand, due to inherent limitations of the data collecting method, data on the diversity and distribution of microfungi and taxa with inconspicuous fruitbodies have remained scarce. Building on the methodological advances provided by next-generation sequencing techniques, we can still greatly improve the exhaustiveness of species inventories by providing complementary information, particularly with regard to asexual fungi and species with microscopic and/or inconspicuous fruiting bodies, in addition to providing new spatial data points for macrofungi already featured in the species lists.

The massive amount of sequence data we generated provide the first kingdom-wide fungal diversity assessment for this coastal ecosystem, offering a complementary view on species diversity with a particularly large contribution on asexual fungi and species with microscopic and/or inconspicuous fruiting bodies that fall beyond the scope of the above mapping project. Even for macrofungi, despite the obvious spatial limitations of the methodology we used, we detected numerous red listed species in our samples, often from previously unknown locations. Several taxa with scarce (<5) previous sporocarp records, such as *Inocybe exilis*, *Pseudobaeospora pyrifera, Russula pascua, Scutellinia vitreola* and several *Tomentella* and *Sebacina* species to name a few, were found in multiple locations, indicating that they may be more widespread in the Netherlands than previously thought (Figure 4). This may be particularly true for *Tomentella*, a species-rich genus with often inconspicuous sporocarps, that is regularly shown to be much more diverse and abundant in soil and root samples than in sporocarp-based diversity assessments (e.g., [15],[75],[76]). We also provide valuable data for additional taxa that are considered either rare or sporadic, often with “vulnerable” or “endangered” red list categories, e.g., *Clavicorona taxophila, Clavulinopsis corniculata*, *C. luteoalba*, *Conocybe subxerophytica*, *Cortinarius alnetorum*, *C. casimiri*, *C. cinnamomeus*, *C. diasemospermus*, *C. parvannulatus*, *Entoloma asprellum*, *E. clandestinum*, *E. longistriatum*, *E. turbidum*, *Geastrum striatum*, *Geoglossum fallax, G. umbratile, Geopora arenicola, Hebeloma collariatum*, *H. leucosarx*, *H. nigellum*, *Hemimycena tortuosa*, *Hydnum rufescens, Hygrocybe helobia*, *H. vitellina*, *Inocybe agardhii*, *I. decipiens*, *I. dunensis, I. griseovelata*, *I. jacobi*, *I. nitidiuscula*, *I. serotina*, *I. vulpinella*, *I. whitei*, *Lactarius aurantiacus*, *Mycena albidolilacea*, *Panaeolus papilionaceus*, *Peziza ampliata, Pleurotus eryngii*, *Russula foetens, Russula puellaris*, *Squamanita odorata*, *Tephrocybe tylicolor*, *Thanatephorus cucumeris*, *Tricholoma lascivum*, *Tulostoma brumale*, *Xerocomus riperiellus* etc.

Highly similar matching sequences from species not formerly reported from the Netherlands represent a special case and confirming the presence of some species will require additional evidence. Some such examples among ascomycetes are the truffle *Tuber anniae*, the earth-tongue *Geoglossum glabrum*, the cup fungus *Geopora cervina*, and the false-morel *Verpa digitaliformis*. In Basidiomycota, the list of taxa formerly not reported from the Netherlands and found among the highly similar (>98%) matching sequences of our OTUs include: *Clavaria californica*, *Cortinarius favrei*, *C. sertipes*, *Heterobasidion parviporum*, *Hygrocybe persistens*, *Inocybe giacomi*, *Mycena simia*, *Pluteus satur*, *Russula integra*, *R. nana*, *Sphaerobolus ingoldii*, and *Tubaria minima*. Occurrence data regarding species that usually occur in arctic-alpine regions, such as *Cortinarius favrei* and *Russula nana*, are particularly interesting, given the geographic position and the lack of mountains in the Netherlands. In the Arctic, these species are symbionts of various dwarf willow species (e.g. *Salix polaris, S. reticulata*) [77],[Geml pers. obs.], while in the Netherlands, they appear to grow with *Salix repens*. Both *Cortinarius favrei* and *Russula nana* have been found associated with *Salix repens* in the oceanic archipelago north of Scotland [26]. It has to be noted that *Russula laccata*, a species originally described from the Netherlands albeit with no current mapping data, is very closely related to *R. nana* and whether or not they are truly distinct species is currently uncertain. Therefore, detailed taxonomic work is needed to test whether or not the two taxa are conspecific and whether *R. laccata* refer to the same taxon as our OTU (no. 695), in which case the name *R. nana* will be preserved based on priority.

## Conclusions

Fungal communities are notoriously difficult to fully characterize for ecological and biodiversity studies and for conservation purposes. Even for macrofungi (e.g., such as mushrooms, true and false truffles etc.), that have the longest history of diversity studies among fungi [1],[70],[78], basic questions about the number of species at a given location or differences in species richness among vegetation types have generally remained unanswered due to taxonomic problems and the scarcity of long-term sporocarp-monitoring projects [78].

Our work provides a wealth of information on the kingdom-wide diversity of fungal communities along the protected coastal dune habitats in the Netherlands, extending both the list of species likely occurring in the Netherlands as well as providing new data points for many rare species. Furthermore, our work highlights that fungal communities found in the rhizosphere of *S. repens* can have very different species composition due to edaphic factors and that the protection of areas that harbour such varied *S. repens* beds is vital for the conservation of co-habiting fungi, including many red-listed species. Future fruitbody collecting projects should utilize our data to concentrate sampling and monitoring efforts on selected localities in order to confirm species occurrences that are currently only based on soil sequence data. Although more detailed, taxon-specific studies will follow, this project already provides examples for the potential contribution of high-throughput soil sequencing studies to fungal mapping and conservation.

### Data Accessability

The raw data have been submitted to the European Nucleotide Archive (ERP001713).

## Supporting Information

Table S1 List of OTUs that showed >97% ITS2 sequence similarity to fully identified fungi in coastal *Salix repens* communities used in this study with best identified match (GenBank or UNITE accession number, Species Hypothesis, name), similarity (%), pairwise alignment length (bp), sequence counts in the sampled sites, phylum, and order classifications.
(XLS)
